# Physical Fitness Training in Patients with Subacute Stroke (PHYS-STROKE): Safety analyses of a randomized clinical trial

**DOI:** 10.1177/17474930211006286

**Published:** 2021-04-07

**Authors:** Torsten Rackoll, Alexander H Nave, Martin Ebinger, Matthias Endres, Ulrike Grittner, Agnes Flöel, ; for the PHYS-Stroke study group

**Affiliations:** 1 Center for Stroke Research Berlin (CSB), Charité – Universitätsmedizin Berlin, Berlin, Germany; 2 NeuroCure Clinical Research Center, Charité – Universitätsmedizin Berlin, Berlin, Germany; 3 Berlin Institute of Health QUEST Center for Transforming Biomedical Research Charité – Universitätsmedizin Berlin, Berlin, Germany; 4 Institute of Neurology, Charité – Universitätsmedizin Berlin, Berlin, Germany; 5 German Center for Cardiovascular Research (DZHK), Partner Site Berlin, Germany; 6 Berlin Institute of Health (BIH), Berlin, Germany; 7 Department of Neurology, Medical Park Berlin Humboldtmühle, Berlin, Germany; 8 German Center for Neurodegenerative Diseases (DZNE), Partner Site Berlin, Berlin, Germany; 9 Institute of Biometry and Clinical Epidemiology, Charité – Universitätsmedizin Berlin, Berlin, Germany; 10 Department of Neurology, University Medicine Greifswald, Greifswald, Germany

**Keywords:** Aerobic treadmill training, subacute stroke, stroke rehabilitation, serious adverse events, safety

## Abstract

**Background and aim:**

To report the six-month safety analyses among patients enrolled in the “Physical Fitness Training in Subacute Stroke—PHYS-STROKE” trial and identify underlying risk factors associated with serious adverse events.

**Methods:**

We performed a pre-specified safety analysis of a multicenter, randomized controlled, endpoint-blinded trial comprising 200 patients with moderate to severe subacute stroke (days 5–45 after stroke) that were randomly assigned (1:1) to receive either aerobic, bodyweight supported, treadmill-based training (n = 105), or relaxation sessions (n = 95, control group). Each intervention session lasted for 25 min, five times weekly for four weeks, in addition to standard rehabilitation therapy. Serious adverse events defined as cerebro- and cardiovascular events, readmission to hospital, and death were assessed during six months of follow-up. Incident rate ratios (IRR) were calculated, and Poisson regression analyses were conducted to identify risk factors for serious adverse events and to test the association with aerobic training.

**Results:**

Six months after stroke, 50 serious adverse events occurred in the trial with a higher incidence rate (per 100 patient-months) in the training group compared to the relaxation group (6.31 vs. 3.22; IRR 1.70, 95% CI 0.96 to 3.12). The association of aerobic training with serious adverse events incidence rates were modified by diabetes mellitus (IRR for interaction: 7.10, 95% CI 1.56 to 51.24) and by atrial fibrillation (IRR for interaction: 4.37, 95% CI 0.97 to 31.81).

**Conclusions:**

Safety analysis of the PHYS-STROKE trial found a higher rate of serious adverse events in patients randomized to aerobic training compared to control within six months after stroke. Exploratory analyses found an association between serious adverse events occurrence in the aerobic training group with pre-existing diabetes mellitus and atrial fibrillation which should be further investigated in future trials.

**Data access statement:**

The raw data and analyses scripts are provided by the authors on a secure online repository for reproduction of reported findings.

## Introduction

The number of stroke survivors with impairments is increasing, rendering effective rehabilitation interventions a major unmet medical need.^1^ Aerobic training is a recommended treatment modality in stroke rehabilitation to counter cardiorespiratory deterioration.^2–4^ However, it remains uncertain whether training in the critical early period of stroke recovery can be carried out safely. Cardiorespiratory stress applied during early rehabilitation might cause adverse effects.^5^

The evidence of safety of aerobic training early after stroke is scarce. The latest Cochrane Collaboration meta-analysis aggregated estimates of adverse effects including cerebro- and cardiovascular events in the stroke population but could not identify a higher risk in aerobic training compared to control interventions.^6^ Of note, the evidence derived mainly from small studies with limited reporting of adverse events.

Surprisingly and in contrast to smaller stroke rehabilitation trials, the results of the recent “Physical Fitness Training in Subacute Stroke” (PHYS-STROKE) trial,^7^ which randomized subacute stroke patients to early aerobic training or relaxation, identified a higher risk of serious adverse events (SAE) within three months post stroke in the training group compared to control.

## Aims

In accordance with the trial protocol, we now report the final safety data of the six-month trial follow-up and additionally provide exploratory analyses aimed to identify patient-related risk factors for SAE associated with aerobic training. The six months’ follow-up was chosen to unveil longer term effects of an early aerobic training in the subacute phase after stroke.

## Methods

The trial protocol,^8^ statistical analysis plan (https://doi.org/10.6084/m9.figshare.5375026.v1), and the primary efficacy endpoints of the multi-center, randomized, controlled PHYS-STROKE trial (clinicaltrials.gov identifier: NCT01953549) were published previously.^7^ The protocol was approved by the local ethics committee of the Charité Universitätsmedizin Berlin (EA1/138/13). Patients with either ischemic or hemorrhagic stroke in the subacute phase (5–45 days post onset) were included into the trial. The inclusion and exclusion criteria of the trial are listed in the Supplementary Table 1. All patients gave written informed consent.

Detailed intervention procedures have been previously described.^7^ In brief, patients were randomly assigned to receive either bodyweight supported, treadmill-based, aerobic training or relaxation sessions, in addition to standard rehabilitation therapy over a period of four weeks with five sessions per week á 25 min each. The target heart rate (THR) in the training group was calculated by the formula: 180 – “years of age”, which was hypothesized to approximate 50–60% of each patient’s maximum heart rate. The THR was reduced by 10 beats per minute in case of β-blocker medication.^9^

Pre-defined safety endpoints included the following SAE: recurrent non-fatal cardio- or cerebrovascular event, readmission to an acute care hospital, or death within six months post stroke. Cerebrovascular event included any stroke or transient ischemic attack confirmed by cerebral imaging with or without clinical manifestation that led to new ICD-10 diagnosis. Readmission to an acute care hospital had to be confirmed by discharge letter.

All SAE occurring during hospitalization at the rehabilitation clinic were monitored by a study site physician and had to be reported within 24 h after onset to the coordinating trial center. SAE that occurred after discharge from hospital were inquired from the trial participants or their relatives and confirmed by discharge letters during clinical follow-up visits at three and six months post stroke. Access to the population registry of Berlin was requested to retrieve current health status for patients unavailable at the six-month follow-up. Medical monitoring appointed by the Center for Stroke Research Berlin checked data fidelity and reporting of SAE at each study site. An independent data and safety monitoring board (DSMB) overlooked all SAE on a regular basis.

As part of the accompanying biomarker study “Biomarkers And Perfusion – Training-Induced Changes After Stroke ” (BAPTISe), cerebral magnetic resonance imaging (MRI) was acquired for a subsample of patients (n = 110) before and after the study intervention.^10^ New ischemic lesions visible on diffusion-weighted imaging with or without clinical manifestation were reported to the coordinating trial center. In an exploratory framework, we analyzed relationships of SAE occurrence with patient characteristics, pre-existing comorbidities, medication, and pertinent blood biomarkers (details in supplementary appendix). All comorbidities needed to have a formal diagnosis issued with the respective ICD code at baseline.

Descriptive summary statistics are presented as mean and standard deviation (SD) or median and interquartile range (IQR) where appropriate. The pre-defined safety endpoints were analyzed using Poisson regression models with individual observation time until six months of follow-up as time at risk, which allows for calculating incidence rates (IR) and incidence rate ratios (IRR) with 95% confidence intervals (CI).^11^ We calculated mixed Poisson regression models with number of SAE as a dependent variable, treatment arm as an independent variable, and a random effect (random intercept) to account for center heterogeneity. Additionally, we incorporated individual observation times (log-transformed time of observation as offset). Sensitivity analysis was performed without recurrent strokes detected on cerebral MRI within the BAPTISe study.

For our exploratory risk factor analysis, we fitted multiple Poisson regression models to assess the association of preexisting comorbidities, treatment group, and SAE. Models were controlled for age, sex, and stroke severity to test robustness of associations. Well-established cardiovascular risk factors, i.e. arterial hypertension, atrial fibrillation, diabetes mellitus, history of cerebrovascular event, history of cardiac disease, number of comorbidities, and related medication (antiplatelets, oral anticoagulants, β-blocker, and statins) were added to the models. Additionally, two-way interaction terms with respective risk factors and treatment group were tested. Decision on the final models was based on Bayesian information criterion (BIC). This was done to achieve parsimonious models with high exploratory power, to avoid overfitting and to avoid inclusion of highly correlated variables. Additionally, to illustrate the direction of interaction effects, we used estimated marginal means of subgroups from the final model. Kaplan-Meier curves were used to verify found associations and to illustrate time to first SAE in relevant risk factor subgroups. All analyses are exploratory with regard to the original analysis plan and were not corrected for multiple testing (see Supplements).

## Results

**Figure 1.**
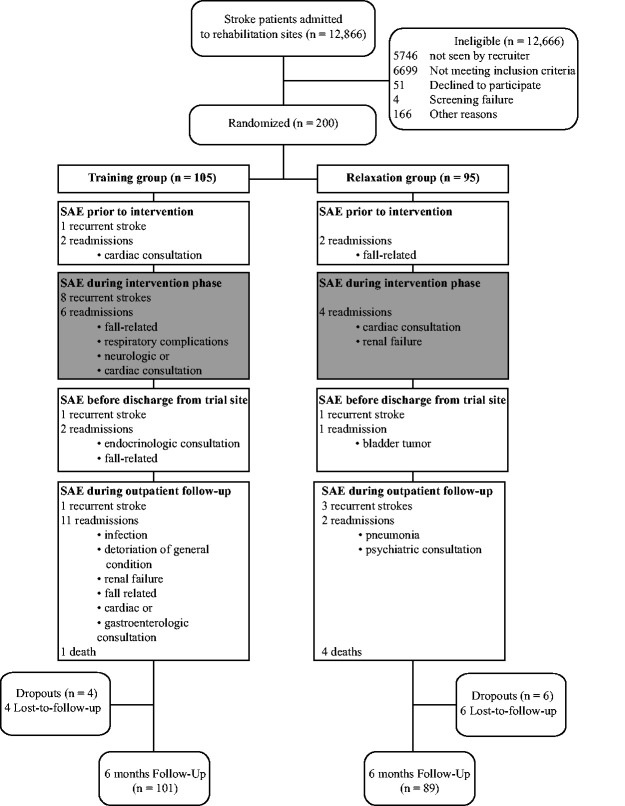
Flow chart of patient enrollment and follow-up of the PHYS-STROKE trial.

The PHYS-STROKE trial randomized 200 patients (training, n = 105 *vs.* relaxation, n = 95) of which 190 (95%) were followed-up until six months post stroke (Figure 1). Four patients of the training group (4%) and six patients of the relaxation group (6%) were lost to follow-up. Eight patients (training, n = 5 vs. relaxation, n = 3) had to discontinue the intervention due to SAE. Protocol adherence and baseline characteristics can be found in Supplementary Tables 2 and 3. Patients had a mean age of 69 years (SD 12 years) and 41% were females. Incident strokes were predominantly ischemic (90%) with a median acute National Institute of Health Stroke Scale (NIHSS) score of 8 (5 to 12). Patients randomly allocated to the training group were more severely affected than patients in the relaxation group (acute NIHSS: 9 (5–12) vs. 7 (5–11)).

Over the course of the trial, 50 SAE occurred in 39 patients. Fifteen recurrent cerebrovascular events and 30 readmissions to an acute care unit were observed. Five patients died within the observation period (training, n = 1 vs. relaxation, n = 4). All fatal events took place after the intervention phase. No cardiovascular event was recorded. Median time from start of intervention to SAE occurrence was 41 days (IQR 14–111). Eighteen SAE (36%) appeared during the intervention phase (training: eight strokes and six readmissions; relaxation: four readmissions) but none during an intervention session. Nine patients (training, n = 6 vs. relaxation, n = 3) had more than one SAE. Individual SAE are listed in the Supplementary Table 4.

Recurrent cerebrovascular events were due to ischemic stroke (n = 13, 10 in the training and 3 in the relaxation group) or transient ischemic attacks (n = 2, one per group). Five ischemic strokes (training, n = 4 vs. relaxation, n = 1) were asymptomatic incidental findings on baseline (n = 2) or post-intervention (n = 3) MRI within the BAPTISe study. Hospital readmissions primarily occurred because of cardiac complications (27%) and are listed in the Supplementary Table 5. In the training group, one patient died due to an urosepsis, and in the relaxation group, two patients died from a recurrent cerebral infarction, one from an acute aortic dissection, and one cause remained unknown. All fatal events were judged unrelated to the intervention by the DSMB. Three recurrent ischemic events in the training group were judged as “possibly although unlikely related to the intervention”; all other SAE were judged “unlikely” to be related to the intervention.

Incidence rates for SAE from Poisson regression analyses are presented in Table 1. Comparing aerobic training with relaxation, within six months of follow-up, the risk per 100 patient-months was 2.3 events and 0.9 events for recurrent cerebrovascular events (IRR: 2.43, 95% CI 0.83 to 8.76, p = 0.13), 4.3 and 2.1 for readmission to an acute hospital (IRR 2.06, 95% CI 0.97 to 4.73, p = 0.07), and 0.11 and 0.93 for fatal events (IRR of 0.22; 95% CI 0.01 to 1.50, p = 0.18), respectively. Sensitivity analysis after exclusion of any incidental MRI findings from the “BAPTISe” substudy (n = 5) demonstrated similar IRR for cerebrovascular events (IRR 2.06, 95% CI 0.58 to 9.59).

**Table 1.** Serious adverse events until six months post-stroke

	All N = 200	Training N = 105	Relaxation N = 95	IRR (Incidence-Rate-Ratio) (95% CI)	*p*
Follow-up time (in days), median [IQR]	153 [139–164]	154 [140–166]	153 [135–163]		
Total SAE IR 95% CI	50 4.60 (1.99–8.53)	33 6.31 (2.89–10.82)	17 3.22 (0.87–6.91)	1.70 (0.96–3.12)	0.07
Cerebrovascular event IR 95% CI	15 1.58 (0.59–2.86)	11 2.25 (1.04–3.88)	4 0.93 (0.29–2.15)	2.43 (0.83–8.76)	0.13
Cardiovascular event	0	0	0	–	–
Readmission to hospital IR 95% CI	30 3.07 (1.45–5.08)	21 4.30 (2.23–6.69)	9 2.08 (1.00–3.75)	2.06 (0.97–4.73)	0.07
Death IR 95% CI	5 0.54 (0.09–1.17)	1 0.11 (0.00–0.89)	4 0.93 (0.19–2.15)	0.22 (0.01–1.50)	0.18

Note: Incidence rates (per 100 patient-months) and incidence rate ratios of serious adverse events between both intervention groups.

Distributions of patient characteristics with SAE occurrence are displayed in the Supplementary Table 6. Unadjusted Poisson regression models identified a higher risk of SAE in patients with vs. patients without arterial hypertension in the training group compared to the relaxation group. Similarly, this was observed for patients with diabetes mellitus (DM), atrial fibrillation (AF), higher hs-CRP, and higher serum cortisol.

**Figure 2.**
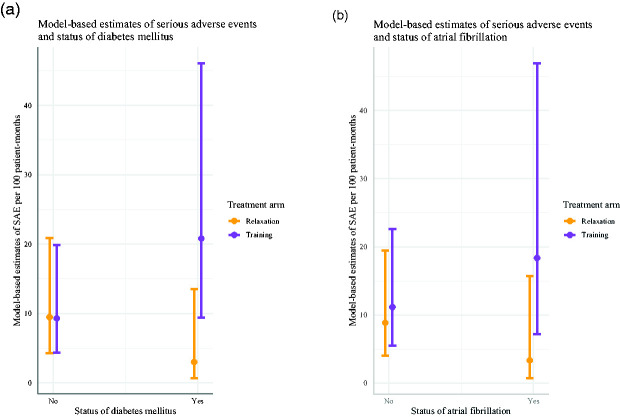
Model-based estimates of events in subgroups of SAE for status of DM (a) and AF (b) in both treatment groups per 100 patient-months. Estimated marginal means are calculated from Poisson regression with Treatment, respective comorbidity, β-blocker medication and an interaction term for treatment with respective comorbidity and are adjusted for age, sex and NIHSS. Results are shown with 95% CI.

**Figure 3.**
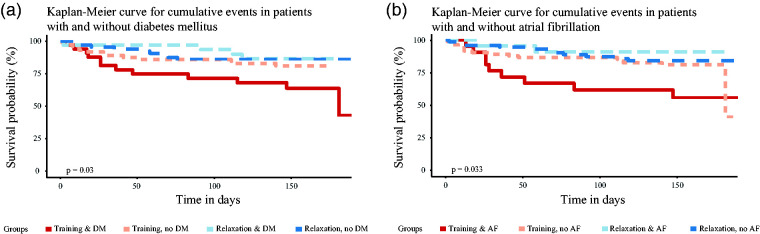
Kaplan–Meier curve of cumulated SAE occurrence stratified by DM (a) and AF (b) status over time. Interactions are shown as treatment with or without comorbidity.

We fitted multiple Poisson regression models with respective preexisting comorbidities and cardiovascular risk constellations and identified parsimonious models with best explanatory power (identified by BIC) analyzing the association of DM and AF with SAE with an interaction between treatment and DM (IRR for interaction 7.10, 95% CI 1.56 to 51.24; Figure 2(a)) and between treatment and AF (IRR for interaction 4.37, 95% CI 0.94 to 31.81; Figure 2(b)), respectively, after adjusting for age, sex, and NIHSS. Kaplan-Meier curves illustrate the course of SAE occurrence for DM and AF in Figure 3. Details of models and model selection can be found in Supplementary Table 7. Due to the small number of SAE and due to collinearities, it was not possible to derive one model including the association of DM and AF with SAE in parallel.

## Discussion

The results of this analysis from a randomized, controlled stroke rehabilitation trial extends the current knowledge of safety of an early bodyweight supported, treadmill-based aerobic training after stroke and provides evidence of potential harms compared to a relaxation program in the early subacute phase after moderate to severe stroke. We detected 50 SAE within six months after stroke which corresponds to a higher incidence rate of SAE than has been reported in previous trials.^6^ Additionally, exploratory analyses identified potential risk factors for SAE. When randomized to the aerobic training group, patients with preexisting diabetes mellitus or atrial fibrillation had a seven-fold or four-fold higher risk of experiencing SAE, respectively. In contrast, risks for SAE were similar for patients without DM or without AF in both intervention groups.

So far, aerobic training was deemed safe in the stroke population.^6^ In our trial, SAE occurred more frequently in the aerobic training group with a peak during the intervention phase. Particularly, recurrent cerebrovascular events happened in temporal relation with the training intervention, whereas patients in the relaxation group had no similar event.

Case fatality rates were lower in the training group after six months of follow-up compared to the relaxation group.

A detailed risk assessment of aerobic training is urgently needed, given that guidance for carers of stroke patients remains contradictory and does not systematically account for individual patient characteristics.^2–4,12,13^ So far, adverse events are rarely reported in stroke rehabilitation trials investigating aerobic training, and associations of adverse events with comorbidities are not discussed.^6^ In fact, only 4 out of 17 trials on aerobic, treadmill-based exercise in the latest Cochrane review reported preexisting comorbidities of study participants in their publications, and in only seven publications, SAE assessment was described.^6^ None of these reports tested for an association of specific patient characteristics and SAE occurrence. The PHYS-STROKE trial, however, reported a possible relation of patient characteristics to SAE occurrence for the first time and aimed to identify subgroups of patients with higher risk.

Diabetic patients are at higher risk for recurrent vascular events and poor outcome after stroke compared to patients without diabetes.^14^ We found a higher association of DM with aerobic exercise in our study population. To the best of our knowledge, no literature exists investigating the risk of aerobic training in stroke patients with DM.

Presence of AF was associated with a higher risk of SAE occurrence in patients undergoing aerobic exercise in the present study. In general, patients with AF demonstrate a high risk of stroke.^15^ Literature on tolerability of aerobic training in stroke patients with AF is scarce. So far, exercise is recommended for all patients with AF, but large exercise intervention studies with assessment on safety are still missing.^16^ In a meta-analysis of exercise rehabilitation trials comprising patients with AF but without stroke, aerobic exercise was considered safe.^17^ The American Association of Cardiovascular and Pulmonary Rehabilitation recommends to slowly progress in exercise intensity in early cardiac rehabilitation.^18^ This may be also applicable in early stroke rehabilitation for patients with concomitant AF, a hypothesis to be explored systematically in future trials.

The PHYS-STROKE trial entails the largest number of subacute stroke patients receiving aerobic training within a randomized clinical trial so far, but several limitations should be considered when interpreting our findings: First, the PHYS-STROKE trial was not powered to detect SAE. However, our trial was the first large study to conduct aerobic training in moderate to severe subacute stroke patients. Further research with rigorous reporting on adverse events is necessary. Second, despite randomization, stroke severity was not balanced between both treatment arms. Patients in the training group had more severe strokes as indicated by the NIHSS, which might have put patients at higher risk for SAE. However, NIHSS score was not substantially associated with SAE occurrence after adjustment for other covariates. Third, we used a pragmatic approach in our trial to assess THR of patients which may have over- or under-estimated the THR that would have been elicited by gradual exercise testing. Therefore, associations between intensity of aerobic training and rate of adverse events should be interpreted with caution. Fourth, the exact mechanisms underlying SAE occurrence following aerobic training cannot be determined in our study. Tentatively, SAE occurrence might also be related to preexisting comorbidities (see Supplementary Tables 3 and 4). However, comorbidities were similarly high in both intervention groups. Fifth, information on risk factors associated with cardiovascular events such as level of previous physical activity or diet were not assessed in the PHYS-STROKE trial. Although data on pre-stroke behavior might be vital, assessment of such measures are subject to recall bias. Lastly, our trial incorporated a rigorous training regimen with training sessions five times per week over a four-week period. A slower progression of training intensity might be necessary to reduce potential risks in a vulnerable patient population.

Aerobic fitness training early after moderate to severe stroke may cause harm compared to relaxation, and increased risk of SAE was particularly attributed to patients with preexisting diabetes mellitus or atrial fibrillation in the PHYS-STROKE trial. Future trials are needed to confirm or refute these exploratory findings.

## Supplemental Material

sj-pdf-1-wso-10.1177_17474930211006286 - Supplemental material for Physical Fitness Training in Patients with Subacute Stroke (PHYS-STROKE): Safety analyses of a randomized clinical trial Click here for additional data file. Supplemental material, sj-pdf-1-wso-10.1177_17474930211006286 for Physical Fitness Training in Patients with Subacute Stroke (PHYS-STROKE): Safety analyses of a randomized clinical trial by Torsten Rackoll, Alexander H Nave, Martin Ebinger, Matthias Endres, Ulrike Grittner, Agnes Flöel and ; for the PHYS-Stroke study group in International Journal of Stroke
