# Supplementary material for: Physical Fitness Training in Patients with Subacute Stroke (PHYS-STROKE): Safety analyses of a randomized clinical trial
Source: Int J Stroke. 2021 Apr 7;17(1):93–100. doi: 10.1177/17474930211006286 (PMC8739607; doi:10.1177/17474930211006286)
Supplement: sj-pdf-1-wso-10.1177_17474930211006286 - Supplemental material for Physical Fitness Training in Patients with Subacute Stroke (PHYS-STROKE): Safety analyses of a randomized clinical trial [file sj-pdf-1-wso-10.1177_17474930211006286.pdf]

Supplementary Appendix

Physical Exercise in Patients with Subacute Stroke (PHYS-STROKE): safety analyses of a randomized clinical trial

Table of contents

Authors and study group 3

Authors 3

Co-Investigators of the PHYS-Stroke study group 3

Supplementary Methods 4

Table 1: Inclusion and exclusion criteria 4

Control intervention 5

Statistics 5

Blood biomarker analysis 5

Data Availability Policy 5

Supplementary Results 6

Table 2: Protocol adherence 6

Table 3: Baseline characteristics 7

Table 4: Characteristics of patients with SAE 9

Table 5: Departments of acute house readmissions, and respective diagnoses (absolute frequencies) 10

Table 6: Baseline comparison of patients with SAE compared to patients without SAE between both intervention groups. 11

Exploratory secondary endpoints 14

Exploratory Risk factor analyses 14

Table 7: Model comparison for association of arterial hypertension, diabetes mellitus, atrial fibrillation and HbA1c with SAE. 15

Authors and study group

Authors

Name	Location	Contribution
Torsten Rackoll, Dipl	Berlin Institute of Health, Berlin	Acquired, analyzed, and interpreted the data; conceptualized and designed the study; performed statistical analysis; drafted the manuscript for intellectual content
Alexander H. Nave, MD	Charité Universitaetsmedizin Berlin	Conceptualized and designed the study; acquired and analyzed the data; drafted the manuscript for intellectual content
Martin Ebinger, MD	Medical Park Humboldtmühle, Berlin	Conceptualized and designed the study; interpreted the data; revised the manuscript for intellectual content
Matthias Endres, MD	Charité Universitaetsmedizin Berlin	Interpreted the data; revised the manuscript for intellectual content
Ulrike Grittner, PhD	Charité Universitaetsmedizin Berlin	Conceptualized and designed the study; supervised the statistical analysis and interpretation of data; revised the manuscript for intellectual content
Agnes Flöel, MD	University Medicine Greifswald, Greifswald	Conceptualized and designed the study; supervised the study; major role in design of the PHYS-Stroke trial; revised the manuscript for intellectual content

Co-Investigators of the PHYS-Stroke study group

Name	Location	Role	Contribution
Andreas Meisel, MD	Charité Universitaetsmedizin Berlin	Chief Executive Director, Center of Stroke Research Berlin	Design and conceptualization of the PHYS-Stroke trial; Fundraising
Stefan Hesse, MD (deceased)	Charité Universitaetsmedizin Berlin	Chair	Design and conceptualization of the PHYS-Stroke trial
Heinrich J. Audebert, MD	Charité Universitaetsmedizin Berlin	Site investigator	Design and conceptualization of the PHYS-Stroke trial
Fabian Klostermann, MD	Charité Universitaetsmedizin Berlin	Site investigator	Site coordination and recruiting
Ursula Müller-Werdan, MD	Evangelisches Geriatriezentrum Berlin Evangelisc	Site investigator	Site coordination and recruiting
Elisabeth Steinhagen-Thiessen, MD	Evangelisches Geriatriezentrum Berlin Evangelisc	Site investigator	Site coordination and recruiting
Holger Bläsing, MD	Median-Klinik Grünheide	Site investigator	Site coordination and recruiting
Anna Gorsler, MD	Kliniken Beelitz GmbH	Site investigator	Site coordination and recruiting
Darius G. Nabavi	Vivantes Klinikum Neukölln	Site investigator	Site coordination and recruiting

Supplementary Methods

Table 1: Inclusion and exclusion criteria

**INCLUSION CRITERIA**	
1.	Diagnosis of stroke (inclusion within 5-45 days after stroke onset); ischemic or haemorrhagic (cortical, subcortical, brainstem), as determined by initial MRI/CT scan of the brain)
2.	Age ≥ 18 years
3.	Able to sit unsupported (i.e. without holding onto supports such as the edge of the bed), with feet supported, for at least 30 seconds
4.	Barthel-Index ≤ 65 at inclusion
5.	Considered able to perform aerobic exercise, as determined by responsible physician
6.	Provision of written informed consent
**EXCLUSION CRITERIA**	
1.	Patient considered unable to comply with study requirements
2.	Stroke due to intracranial haemorrhage primarily due to bleeding from ruptured aneurysm or arteriovenous malformation
3.	Progressive stroke
4.	Unable to perform the required exercises due to a) medical, b) musculo-skeletal, or c) neurological problems (for details see below, 4a-c)
4a.	Medical problems: unstable cardiovascular condition, or other serious cardiac conditions (e. g., New York Heart Association criteria for Class IV heart disease, hospitalization for myocardial infarction or heart surgery within 120 days, severe cardiomyopathy or documented serious and unstable cardiac arrhythmias)
4b.	Musculoskeletal problems: restricted passive range of motion in the major lower limb joints (i.e. an extension deficit of > 20° for the affected hip or knee joints, or a dorsiflexion deficit of > 20° for the affected ankle)
4c.	Neurological problems: severity of stroke-related deficits
5.	Required help of at least 1 person to walk before stroke due to neurological (e. g., advanced Parkinson's disease, Amyotrophic Lateral Sclerosis, Multiple Sclerosis) or non-neurological co-morbidities (e. g. heart failure, orthopaedic problems)
6.	Life expectancy of less than 1 year as determined by responsible physician
7.	Drug or alcohol addiction within the last six months
8.	Significant current psychiatric illness defined as medication-refractory of bipolar affective disorder, psychosis, schizophrenia or suicidality.
9.	Current participation in another interventional trial

MRI = magnetic resonance imaging | CT = computer tomography

Control intervention

The active control group received relaxation sessions for the same duration as the training group. Relaxation focused on contraction and relaxation of muscle groups of the upper body. In both intervention groups, the heart rate of patients was monitored throughout each session.

Statistics

As exploratory secondary safety analyses, we analyzed the influence of SAE on previously reported primary efficacy endpoints, hospitalization time and discharge to aftercare facility. Additional exploratory analyses were performed to explore the influence of SAE occurrence on previously reported efficacy endpoints. Linear mixed effects regression was used to calculate the difference in hospitalization time and multinomial regression was used to estimate odds ratios (OR) for discharge to aftercare. In post-hoc analyses, blood biomarkers were used to test if associated comorbidities in risk factor analyses were adequately controlled. Statistical tests presented in the main manuscript as well as in the supplemental material were done in R statistical software version 3.6.2 with the 'tableone'[14] and the 'lme4'[15] package.

Blood biomarker analysis

The following blood-derived biomarkers were analyzed using standard procedures and are included as part of participants' baseline characterization in the supplements: Hemoglobin, hematocrit, erythrocytes, leukocytes, thrombocytes, glucose, insulin, HbA1c, high density lipoprotein, low density lipoprotein, triglycerides, lipoprotein a, kidney, creatinine, estimated glomerular filtration rate (eGFR), fibrinogen, high-sensitivity C-reactive protein (hs-CRP), tumor necrosis factor alpha (TNF-alpha), interleukin 6 (IL-6), cortisol, and thyroid-stimulating hormone (TSH).

Fibronogen:

The quantitative determination of fibrinogen levels was performed based on the Clauss method in citrated blood plasma by using the HemosIL® Q.F.A. Thrombin (Bovine) kit, Instrumentation Laboratories.

CRP, TnF-a, IL-6:

Blood levels of high-sensitive C-reactive protein (hs-CRP), tumor necrosis factor-alpha (TNF α), and interleukin-6 (IL-6) were quantitatively determined using solid-phase, chemiluminescent immunometric assays (IMMULITE® 1000, Siemens Healthcare Diagnostics).

Insulin:

Serum insulin levels were quantified with a electrochemiluminescence immunoassay "ECLIA" using cobas® Elecsys immunoassay systems, Roche Diagnostics.

Lp(a):

Lipoprotein(a) was quantified by kinetic nephelometry (Immage Immunchemie System, Beckmann Coulter) using a polyclonal antibody in an assay insensitive to apo(a) isoforms.

Data Availability Policy

The raw data and analyses scripts are provided by the authors on a secure online repository for reproduction of reported findings (https://doi.org/10.5281/zenodo.3899830). Data include anonymized individual patient variables for results reported here, and analyses scripts used in this study. Data will be available with the publication of the article. Further information can be shared on individual request addressed to the corresponding author.

Supplementary Results

Table 2: Protocol adherence

**Intervention**	**Training**<br>**N = 105**	**relaxation**<br>**N = 95**
Number of performed intervention sessions, mean (SD)	16 (6)	17 (5)
Duration of core intervention in minutes, mean (SD)	21 (4)	24 (3)
Heart rate delta in bpm, mean (SD)	15 (9)	-2 (3)
Blood pressure pre-session in mmHg, mean (SD)	126/75 (11/9)	126/74 (12/8)
Blood pressure post-session in mmHg, mean (SD)	128/76 (12/9)	123/73 (12/8)
Standard care physiotherapy received, median [IQR]	2220 [1545 – 2782]	2122 [1540 – 2692]

Comparing the frequencies of intervention sessions in which THR was achieved between patients with and without SAE, no difference between groups was detected (SAE: median 88.9% [IQR 27 to 100] vs. No SAE: median 70% [IQR 27 to 100]).

Table 3: Baseline characteristics

Baseline characteristics of participants stratified by trial intervention group.*

	**Aerobic fitness training group** N = 105	**Relaxation group** N = 95	**Study population** N = 200
Age in years, mean (SD)	69 (12)	70 (11)	69 (12)
Female sex, n (%)	45 (43)	36 (38)	81 (41)
NIHSS score, median [IQR]‡	9 [5 – 12]	7 [5 – 11]	8 [5 – 12]
Left hemisphere stroke, n (%)	40 (38)	48 (50)	88 (44)
Anterior circulation stroke, n (%)	84 (80)	72 (76)	156 (78)
Ischaemic stroke, n (%)	91 (87)	90 (95)	181 (91)
Treatment with alteplase, n (%)§	34 (37)	27 (30)	61 (34)
Ischemic stroke aetiology§			
LAA, n (%)§	17 (19)	19 (21)	36 (20)
Cardioembolism, n (%)§	18 (20)	18 (20)	36 (20)
Small vessel occlusion, n (%)§	16 (18)	15 (17)	31 (17)
Other aetiology, n (%)§	3 (3)	4 (4)	7 (4)
Undetermined aetiology, n (%)§	34 (37)	28 (31)	62 (34)
Competing etiologies, n (%)§	3 (3)	6 (7)	9 (5)
**Pre-existing comorbidities**			
Atrial fibrillation, n (%)	23 (22)	23 (24)	46 (23)
Diabetes mellitus, n (%)	32 (31)	31 (33)	63 (32)
Arterial hypertension, n (%)	86 (82)	80 (84)	166 (83)
Hypercholesterolemia, n (%)	43 (41)	37 (39)	80 (40)
History of cerebrovascular event, n(%) [a]	27 (26)	27 (28)	54 (27)
History of cardiovascular disease, n (%) [b]	13 (12)	21 (22)	34 (17)
No. of comorbidities, n (%) [c]			
0	3 (3)	5 (5)	8 (4)
1 – 3	68 (65)	58 (61)	126 (63)
> 3	34 (32)	32 (34)	66 (33)
Smoking‖	18 (17)	11 (12)	29 (15)
**Clinical parameters**			
Heart rate in beats per minute, mean (SD)	77 (12)	76 (14)	76 (13)
Systolic blood pressure in mmHg, mean (SD)	127 (18)	131 (18)	129 (18)
Diastolic blood pressure in mmHg, mean (SD)	73 (13)	77 (13)	75 (13)
Body mass index in kg/m^2, mean (SD)	26 (4)	26 (4)	26 (4)
**Concomitant medication**			
Antiplatelets, n (%)	48 (46)	54 (57)	102 (51)
Oral anticoagulation, n (%)	57 (54)	44 (46)	101 (51)
Beta blocker, n (%)	49 (47)	47 (50)	96 (48)
Statins, n (%)	79 (75)	80 (84)	159 (80)

* Patients in the aerobic training group received physical fitness training plus standard care. Patients in the relaxation group received relaxation sessions plus standard care.

† No data available in four patients, because patients were excluded as screening failures prior to intervention start.

‡ Scores on the National Institutes of Health Stroke Scale (NIHSS) range from 0 to 42, with higher scores indicating greater stroke severity. Assessed on day 3-5 after stroke. The NIHSS score of one patient was missing due to missing charts from the acute hospital.

§ Reported proportions of patients treated with alteplase and proportions of stroke aetiology refer only to ischemic stroke patients.

‖ History of smoking was not available in 25 patients.

[a] Cerebrovascular event comprises any occurrence of either stroke or transient ischemic attack

[b] Cardiovascular disease comprises any occurrence of either coronary artery disease, myocardial infarction or periphery artery disease

[c] Number of comorbidities reflects the load of comorbidities and comprises the number of all documented pre-existing conditions as atrial fibrillation, diabetes mellitus, arterial hypertension, coronary artery disease, transient ischemic attack, stroke, coagulation disorder, periphery artery disease, myocardial infarction, migraine, thyroid disease, tumor, sleep apnoe, amyotrophic lateral sclerosis, multiple sclerosis, morbus Parkinson, hypercholesterolemia, or any other disease.

Table 4: Characteristics of patients with SAE

Treatment arm	Patient	Type of first SAE	Type of second SAE	Type of third SAE	Time from stroke to intervention start in days	Atrial fibrillation	Diabetes mellitus	Arterial hypertension	History of cerebrovascular	History of cardiovascular	Hypercholesterolemi
Relaxation	1	Cerebrovascular event	Readmission to hospital		10			X		X	
	2	Cerebrovascular event			14			X		X	X
	3	Cerebrovascular event			22			X			
	4	Cerebrovascular event			24		X	X	X		X
	5	Readmission to hospital	Readmission to hospital		NA		X				
	6	Readmission to hospital			40			X			
	7	Readmission to hospital			43	X					
	8	Readmission to hospital			40	X			X		X
	9	Readmission to hospital	Readmission to hospital	Death	21			X	X	X	
	10	Readmission to hospital			9			X	X		X
	11	Death			8						
	12	Death			28		X	X		X	
	13	Death			13		X	X			X
Training	14	Cerebrovascular event	Readmission to hospital	Cerebrovascular event	26			X	X		
	15	Cerebrovascular event	Readmission to hospital		17	X	X	X		X	X
	16	Cerebrovascular event			19		X	X			X
	17	Cerebrovascular event			39	X	X	X			
	18	Cerebrovascular event			53			X		X	
	19	Cerebrovascular event			15	X	X	X	X		X
	20	Cerebrovascular event			37		X	X			
	21	Cerebrovascular event	Readmission to hospital		8			X	X		X
	22	Cerebrovascular event			9			X			X
	23	Readmission to hospital	Readmission to hospital		34	X	X	X	X		
	24	Readmission to hospital			17	X		X	X		
	25	Readmission to hospital			52		X	X		X	
	26	Readmission to hospital			41			X			
	27	Readmission to hospital			47			X			X
	28	Readmission to hospital			39	X		X			
	29	Readmission to hospital			47		X	X	X	X	
	30	Readmission to hospital			28	X					
	31	Readmission to hospital	Readmission to hospital		17	X	X	X			
	32	Readmission to hospital			39	X	X	X	X		X
	33	Readmission to hospital			NA		X	X			
	34	Readmission to hospital			12		X	X			X
	35	Readmission to hospital			14			X			
	36	Readmission to hospital			8						
	37	Readmission to hospital	Cerebrovascular event		11		X	X	X		X
	38	Readmission to hospital			16			X			
	39	Death			24			X		X	X

Table 5: Departments of acute house readmissions, and respective diagnoses (absolute frequencies)

Hospital ward	Aerobic fitness training		Relaxation	
	No. of participants experienced event	Total number of events	No. of participants experienced event	Total number of events
**Cardiology**	**7**	**7**	**2**	**2**
Mitral regurgitation		1		0
No new diagnosis		1 *		0
Tachy-Brady Arrhythmia		1		0
Implantation of cardiac pacemaker		1		1
New diagnosis of atrial fibrillation		2 (1*)		0
Cardiac decompensation with pleural effusions		1		0
Ventricular tachycardia		0		1
**Neurology**	**5**	**5**	**0**	**0**
Traumatic subdural haematoma		1		0
Extra-intracranial bypass		1		0
Confirmation of tandem stenosis of the A. basilaris		1		0
New stroke (incidental finding)		1		0
Giant-cell arteritis		1		0
**Gastroenterology**	**3**	**3**	**0**	**0**
Urinary tract infection with Hyponatremia		1		0
Refusal to ingest food		1		0
Ischaemic colitis		1		0
**Orthopaedics / Traumatology**	**2**	**2**	**2**	**2**
Femoral neck fracture		1		2 *
Metatarsal fracture		1		0
**Pneumology**	**1**	**1**	**1**	**1**
Respiratory insufficiency		1		0
Pneumonia		0		1
**Nephrology**	**1**	**1**	**2**	**2**
Kidney failure		1		2
**Endocrinology**	**1**	**1**	**0**	**0**
Suspected insulinoma		1		0
**Other**	**1**	**1**	**0**	**0**
Deterioration of general condition		1		0
**Tumor center**	**0**	**0**	**1**	**1**
Bladder carcinoma		0		1
**Psychiatry**	**0**	**0**	**1**	**1**
Suicidal tendency		0		1
**Surgery**	**1**	**1**	**0**	**0**
Wound erysipelas		1		0

* SAE prior to intervention

Table 6: Baseline comparison of patients with SAE compared to patients without SAE between both intervention groups.

Baseline characteristics	Training		Relaxation		IRR (95% CI) for interaction of treatment group and characteristic ‡
	No SAE	SAE	No SAE	SAE	
**Socio-demographics**	N = 79	N = 26	N = 82	N = 13	
Age in years, mean (SD)	68 (13)	72 (9)	70 (11)	72 (9)	1.00 (0.94 to 1.05)
Female sex, no. (%)	31 (39)	14 (54)	29 (35)	7 (54)	0.58 (0.16 to 1.92)
**Initial stroke**					
Time since stroke to intervention start in days, median [IQR] #	31 [17 to 37]	24 [15 to 39]	28 [18 to 42]	22 [12 to 31]	1.02 (0.98 to 1.08)
Ischaemic stroke, no. (%)	66 (84)	25 (96)	77 (94)	13 (100)	-
Left hemisphere stroke, no. (%)	34 (43)	6 (23)	45 (55)	3 (23)	2.35 (0.62 to 10.15)
Anterior circulation, no. (%)	20 (25)	5 (19)	18 (22)	4 (31)	0.34 (0.09 to 1.31)
NIHSS, mean (SD) §	9.3 (4.7)	8.4 (5.4)	8.3 (4.4)	8.5 (4.7)	0.98 (0.86 to 1.13)
**Preexisting comorbidities**					
Atrial fibrillation, no (%)	14 (18)	9 (35)	21 (26)	2 (15)	4.18 (0.94 to 29.82)
Diabetes mellitus, no (%)	19 (24)	13 (50)	27 (33)	4 (31)	2.67 (0.79 to 10.05)
Arterial hypertension, no (%)	62 (79)	24 (92)	71 (87)	9 (69)	7.93 (1.47 to 61.42)
History of cerebrovascular diseases, no (%) [a]	19 (24)	8 (31)	23 (28)	4 (31)	1.21 (0.36 to 4.30)
History of cardiovascular disease, no (%) [b]	8 (10)	5 (19)	17 (21)	4 (31)	0.63 (0.16 to 2.35)
No. of comorbidities, no. (%) [c]					
0	3 (4)	0 (0)	4 (5)	1 (8)	[d]
1 – 3	55 (70)	13 (50)	49 (60)	9 (69)	0.49 (0.14 to 1.62)
> 3	21 (27)	13 (50)	29 (35)	3 (23)	2.29 (0.68 to 8.62)
History of smoking (%) |	13 (17)	5 (20)	10 (12)	1 (8)	2.52 (0.39 to 49.79)

**Clinical parameters**					
Heart rate in beats per minute, mean (SD)	78 (12)	73 (13)	77 (14)	73 (13)	0.97 (0.92 to 1.02)
Systolic blood pressure in mmHg, mean (SD)	126.1 (17.5)	128.3 (18.2)	130.8 (18.9)	133.5 (15.6)	1.01 (0.98 to 1.04)
Diastolic blood pressure in mmHg, mean (SD)	73.6 (13.6)	71.9 (9.0)	77.0 (13.3)	75.2 (14.5)	1.02 (0.97 to 1.07)
Body mass index in kg/m^2, mean (SD)	25.8 (4.3)	26.4 (4.3)	26.0 (4.2)	25.6 (4.2)	1.01 (0.89 to 1.15)
**Pharmaceutical agents, no (%)**					
Antiplatelets	36 (47)	12 (50)	46 (59)	8 (73)	0.87 (0.22 to 3.18)
Anticoagulation	37 (49)	20 (83)	39 (50)	5 (46)	3.12 (0.72 to 15.16)
Statins	55 (72)	20 (83)	67 (86)	9 (82)	4.57 (0.91 to 23.30)
β-blocker	30 (40)	16 (67)	39 (50)	6 (55)	1.75 (0.43 to 6.89)
**Blood draw**					
**Full blood count**					
Hemoglobin, in mmol/l, median [IQR][1]	8.60 [7.80 to 9.22]	8.15 [7.60 to 8.70]	8.30 [7.70 to 9.20]	8.10 [6.90 to 9.10]	1.23 (0.72 to 2.10)
Hematocrit, in %, median [IQR][1]	40.50 [37.80 to 43.32]	39.00 [36.42 to 41.95]	39.70 [36.60 to 42.90]	36.60 [32.90 to 41.30]	1.04 (0.92 to 1.19)
Erythrocytes, in Tpt/l, median [IQR][1]	4.63 [4.23 to 4.89]	4.42 [4.02 to 4.78]	4.53 [4.14 to 4.90]	4.40 [4.10 to 4.87]	1.15 (0.42 to 3.15)
Leukocytes, in Gpt/l, median [IQR][1]	7.00 [5.88 to 8.22]	7.45 [5.85 to 8.75]	7.20 [6.30 to 8.70]	7.00 [6.00 to 9.00]	0.88 (0.65 to 1.20)
Thrombocytes, in Gpt/l, median [IQR][1]	280 [244 to 334]	253 [192 to 278]	275 [208 to 337]	260 [236 to 308]	1.00 (0.99 to 1.00)
**Metabolic parameters**					
Glucose, in mmol/l, median [IQR][2]	6.05 [5.38 to 6.96]	6.88 [5.99 to 8.99]	6.05 [5.38 to 7.49]	7.22 [5.66 to 8.83]	1.05 (0.84 to 1.36)
Insulin, in µU/ml, median [IQR][1]	9.95 [7.05 to 14.43]	12.15 [7.67 to 17.60]	9.10 [7.20 to 14.50]	9.00 [5.80 to 12.90]	1.03 (0.98 to 1.11)
HbA1c, in %, median [IQR][1]	5.75 [5.40 to 6.32]	5.95 [5.53 to 6.70]	5.80 [5.50 to 6.50]	5.80 [5.30 to 6.40]	1.95 (0.94 to 4.56)
High density lipoprotein, in mmol/l, median [IQR][3]	1.20 [0.96 to 1.53]	1.29 [1.17 to 1.47]	1.17 [0.96 to 1.43]	1.12 [1.07 to 1.43]	0.32 (0.06 to 1.71)
Low density lipoprotein, in mmol/l, median [IQR][3]	2.30 [1.70 to 3.00]	1.95 [1.70 to 2.63]	2.10 [1.50 to 2.50]	1.90 [1.60 to 2.20]	0.68 (0.37 to 1.29)
Triglycerides, in mmol/l, median [IQR][3]	1.31 [1.05 to 1.68]	1.14 [1.00 to 1.72]	1.30 [1.07 to 1.68]	1.28 [0.80 to 1.72]	1.01 (0.31 to 3.49)

Lipoprotein a, in mg/dl, median [IQR][4]	24.40 [9.77 to 66.12]	43.65 [12.55 to 78.65]	22.80 [9.45 to 58.70]	23.00 [5.25 to 59.80]	1.00 (0.99 to 1.01)
**Kidney**					
Creatinine, in µmol/l, median [IQR][3]	80.00 [68.00 to 89.00]	84.00 [70.50 to 100.00]	79.00 [68.00 to 92.00]	81.00 [69.00 to 96.00]	0.99 (0.99 to 1.00)
eGFR, in ml/min, median [IQR][3]	71.00 [58.00 to 94.00]	62.50 [53.50 to 72.25]	69.00 [55.00 to 94.00]	75.00 [52.00 to 82.00]	1.00 (0.98 to 1.03)
**Inflammation**					
Fibrinogen, in g/l, median [IQR][5]	3.94 [3.10 to 5.01]	4.32 [3.33 to 4.99]	3.87 [3.38 to 4.73]	3.67 [3.07 to 4.06]	1.80 (0.94 to 3.76)
hs-CRP, in mg/l, median [IQR][6]	5.21 [1.66 to 12.90]	5.58 [1.52 to 16.85]	6.26 [2.04 to 13.22]	4.82 [0.98 to 8.38]	1.06 (1.00 to 1.16)
TNF-alpha, in pg/ml, median [IQR][7]	7.70 [6.30 to 10.15]	9.05 [7.65 to 10.50]	9.00 [7.00 to 10.95]	8.45 [6.68 to 11.20]	1.01 (0.88 to 1.17)
IL-6, in pg/ml, median [IQR][8]	3.75 [2.82 to 5.78]	6.05 [3.40 to 8.67]	4.40 [3.00 to 7.35]	4.60 [2.67 to 5.72]	1.04 (0.96 to 1.22)
**Hormones**					
Cortisol, in nmol/l, median [IQR][3]	163 [130 to 195]	175 [148 to 233]	177 [142 to 208]	154 [134 to 184]	1.01 (1.00 to 1.02)
TSH, in µlU/ml, median [IQR][9]	1.44 [0.86 to 2.10]	2.10 [0.85 to 3.47]	1.38 [0.88 to 2.07]	1.79 [1.23 to 2.36]	0.86 (0.65 to 1.26)

Patients in the training group received physical fitness training plus standard care. Patients in the relaxation group received relaxation sessions plus standard care. eGFR denotes estimated Glomerular Filtration Rate, hs-CRP denotes high-sensitivity C-reactive protein, TNF-alpha denotes Tumor Necrosis Factor alpha, IL-6 denotes Interleukin 6 and TSH denotes Thyroid-stimulating hormone. Incidence Rate Ratios are calculated from unadjusted Poisson regression models with Treatment, patient characteristics and an interaction term of treatment arm with respective patient characteristic.

‡ IRR indicating differential Incidence of SAE in treatment groups by characteristic.

# Data are missing for four patients due to SAE prior to first day of intervention.

§ Data are missing for one patient in the relaxation group due to missing charts from the acute hospital.

| History of smoking was not available in 25 patients.

[a] Cerebrovascular event comprises any occurrence of either stroke or transient ischemic attack.

[b] Cardiovascular event comprises any occurrence of either coronary artery disease, myocardial infarction or periphery artery disease.

[c] Number of comorbidities reflects the load of comorbidities and comprises the number of all documented pre-existing conditions as atrial fibrillation, diabetes mellitus, arterial hypertension, coronary artery disease, transient ischemic attack, stroke, coagulation disorder, periphery artery disease, myocardial infarction, migraine, thyroid disease, tumor, sleep apnoe, amyotrophic lateral sclerosis, multiple sclerosis, morbus Parkinson, hypercholesterolemia, or any other disease.

[d] Unable to compute IRR due to low number of cases.

[1] Data are missing for three patients in the training group and for one in the relaxation group.

[2] Data are missing for five patients in the training group and for one in the relaxation group.

[3] Data are missing for two patients in the training group and for one in the relaxation group.

[4] Data are missing for seven patients in the training group and for eight in the relaxation group.

[5] Data are missing for five patients in the training group and for two in the relaxation group.

[6] Data are missing for six patients in the training group and for two in the relaxation group.

[7] Data are missing for four patients in the training group and for three in the relaxation group.

[8] Data are missing for 19 patients in the training group and for 15 in the relaxation group.

[9] Data are missing for five patients in the training group and for four in the relaxation group.

Exploratory secondary endpoints

Mean hospitalization time was 67 (SD 31) days in both groups and the amount of applied physiotherapy (median 2220 [1545 – 2782] min *vs.* and 2122 [1540 – 2692] min, respectively) was similar in both groups. Hospitalization times at the rehabilitation clinic of both treatment groups showed differences between patients with SAE and patients without SAE (training: 6, 95% CI -10 to 23 days *vs.* relaxation: 22, 95% CI -11 to 56 days). Relative to being released home, patients with an SAE during hospital stay were less likely to be discharged to a nursing home as patients without SAE (OR 0.55, 95% CI 0.07 to 4.50) and more likely to be discharged to another rehabilitation clinic (OR 1.75, 95% CI 0.19 to 15.77). Exploratory analysis revealed that SAE occurrence was not associated with the primary efficacy endpoint maximal walking speed three months after stroke (0.0 m/s 95% CI -0.2 to 0.2) or six months after stroke (0.0 m/s, 95% CI -0.2 to 0.2). In contrast, occurrence of SAE was associated with a lower Barthel-Index (-11 points, 95% CI -19 to -3) at three months, but this association was weaker at six months after stroke (-5 points, 95% CI -12 to 1).

Exploratory Risk factor analyses

The final adjusted model analyzing the association of DM with SAE included treatment arm, DM, β-blocker medication, and the interaction between treatment arm and DM diagnosis (IRR for interaction 7.10, 95% CI 1.56 to 51.24). Post-hoc estimated marginal means of interaction terms equally demonstrated a higher IRR in patients with DM (6.96, 95% CI 1.60 to 30.35) compared to patients without DM (0.98, 95% CI 0.45 to 2.16) when comparing training and control groups. When diabetes mellitus was substituted by HbA1c in the model, a higher risk of SAE was similarly observed in the training group (IRR for interaction: 3.52, 95% CI 1.41 to 9.29).

With regard to AF, the final model analyzing the association with SAE occurrence included treatment, AF, β-blocker medication, and the interaction between treatment arm and AF (IRR for interaction 4.37, 95% CI 0.94 to 31.81). Observed associations remained stable after controlling for age, sex, and NIHSS. Post-hoc calculation of estimated marginal means showed a higher risk in patients with AF (IRR 5.50, 95% CI 1.22 to 24.80) compared to patients without AF (IRR 1.26, 95% CI 0.60 to 2.66) when comparing training and control group.

Table 7: Model comparison for association of arterial hypertension, diabetes mellitus, atrial fibrillation and HbA1c with SAE.

	**Model 1** (model with treatment, hypertension and interaction of treatment and hypertension), Incidence-Rate-Ratio (95%CI)	**Model 2** (model with treatment, diabetes and interaction of treatment and diabetes), Incidence-Rate-Ratio (95%CI)	**Model 3** (model with treatment, AF and interaction of treatment and AF), Incidence-Rate-Ratio (95%CI)	**Model 2.1** (model with treatment, HbA1c and interaction of treatment and HbA1c), Incidence-Rate-Ratio (95%CI)
Intercept	0.03 (0.01 to 0.07) ***	0.02 (0.01 to 0.04) ***	0.02 (0.01 to 0.04) ***	0.01 (0.00 to 0.02) ***
Treatment arm training group	0.44 (0.06 to 2.68)	0.98 (0.44 to 2.19)	1.26 (0.60 to 2.72)	3.02 (1.38 to 7.75) *
Arterial hypertension	0.51 (0.16 to 2.46)			
Diabetes mellitus		0.32 (0.05 to 1.18)		
Atrial fibrillation			0.38 (0.06 to 1.42)	
HbA1c in %				0.27 (0.11 to 0.62) **
Age in years	1.01 (0.97 to 1.04)	1.01 (0.98 to 1.05)	1.01 (0.98 to 1.04)	1.01 (0.98 to 1.04)
Female sex	1.99 (1.06 to 3.69) *	1.90 (1.04 to 3.50) *	1.74 (0.94 to 3.24)	1.73 (0.92 to 3.28)
Stroke severity (NIHSS)	0.98 (0.91 to 1.06)	0.99 (0.92 to 1.07)	0.99 (0.92 to 1.07)	1.00 (0.92 to 1.08)
Beta blocker medication	2.45 (1.26 to 5.07) *	2.27 (1.17 to 4.70) *	2.47 (1.26 to 5.12) *	3.07 (1.52 to 6.62) **
History of smoking				
Waist-to-hip ratio				
Interaction arterial hypertension with treatment group	5.30 (0.76 to 45.55)			
Interaction diabetes with training group		7.10 (1.56 to 51.24) *		
Interaction atrial fibrillation with training group			4.37 (0.94 to 31.81)	
Interaction HbA1c with training group				3.52 (1.41 to 9.29) **
N	188	188	188	184
Pseudo R^2 (Fixed effects)	24.1%	25.8%	20.9%	49.6%
Pseudo R^2 (total)	§	§	§	53.1%
AIC	244.8	240.6	244.5	233.7
BIC	273.9	269.7	273.7	262.6

*** $p < 0.001$; ** $p < 0.01$; * $p < 0.05$

§ Computation of random effect variances not possible because some variance equal to zero.

Table 7 continued (adjustment of Model 2 and 3 for patient-related lifestyle risk factors).

	**Model 2.2** (model with treatment, diabetes and interaction of treatment and diabetes adjusted for lifestyle risk factors), Incidence-Rate-Ratio (95%CI)	**Model 3.1** (model with treatment, AF and interaction of treatment and AF adjusted for lifestyle risk factors), Incidence-Rate-Ratio (95%CI)
Intercept	0.02 (0.01 to 0.04)*	0.02 *** (0.01 to 0.04)
Treatment arm training group	1.04 (0.46 to 2.32)	1.26 (0.59 to 2.75)
Arterial hypertension		
Diabetes mellitus	0.31 (0.05 to 1.16)	
Atrial fibrillation		0.41 (0.06 to 1.55)
HbA1c in %		
Age in years	1.01 (0.98 to 1.05)	1.01 (0.98 to 1.04)
Female sex	2.03 (1.00 to 4.02) *	1.94 (0.95 to 3.90)
Stroke severity (NIHSS)	0.98 (0.91 to 1.06)	0.99 (0.91 to 1.06)
Beta blocker medication	2.23 (1.13 to 4.65)*	2.29 * (1.15 to 4.80)
History of smoking	0.87 (0.29 to 2.12)	0.87 (0.29 to 2.10)
Waist-to-hip ratio	4.99 (0.06 to 354.23)	8.45 (0.10 to 589.46)
Interaction arterial hypertension with treatment group		
Interaction diabetes with training group	6.18 (1.33 to 45.20) *	
Interaction atrial fibrillation with training group		4.18 (0.88 to 29.22)
N	187	187
Pseudo R² (Fixed effects)	24.3%	20.3%
Pseudo R² (total)	§	§
AIC	237.9	240.3
BIC	273.5	275.8
